# The Function of Glial Cells in the Neuroinflammatory and Neuroimmunological Responses

**DOI:** 10.3390/cells11040659

**Published:** 2022-02-14

**Authors:** Ruqayya Afridi, Makoto Tsuda, Hoon Ryu, Kyoungho Suk

**Affiliations:** 1 Department of Pharmacology, School of Medicine, Kyungpook National University, Daegu 41944, Korea; r.afridi36@gmail.com; 2 BK21 Plus KNU Biomedical Convergence Program, Department of Biomedical Sciences, School of Medicine, Kyungpook National University, Daegu 41944, Korea; 3 Department of Molecular and System Pharmacology, Graduate School of Pharmaceutical Sciences, Kyushu University, Fukuoka 812-8582, Japan; tsuda@phar.kyushu-u.ac.jp; 4 Center for Neuroscience, Brain Science Institute, Korea Institute of Science and Technology (KIST), Seoul 02792, Korea; hoonryu@kist.re.kr; 5 Brain Science and Engineering Institute, Kyungpook National University, Daegu 41944, Korea

The historical concept of glia just as the glue of brain tissue has been challenged by the accumulation of concrete evidence showing the multifunctional role of these cells during development and in the adult brain [1]. The brain tissue is densely populated by many glial cell types, each having a unique molecular signature and specialized functions in the central nervous system (CNS) [2]. These functions include providing metabolic support to neurons, maintaining fast impulse conduction, modulating synaptic formation, maturation, and function, and regulating parenchymal homeostasis [2]. Additionally, a subset of the glial population, including microglia and astrocytes, is also specialized in immune functions and protects the brain tissue. Recent works in the neuroscience field have advanced our understanding of glial biology, and it is now known that all neurological diseases have some glial component, appearing either as a primary or secondary disease cause. The collection of articles in this Special Issue (SI) provided deeper insights into the gliocentric view of neuroimmune disorders of the nervous system.

In addition to the major glial cell types, including astrocytes, microglia, and oligodendrocytes, many region-specific glial cells populate the brain tissue. Bergmann glia (BG) are the principal glia in the cerebellum, outnumbering neurons [3]. These cells are vital for the migration and correct layering of granule cells in early cerebellar development [3]. Cerebellar cortical dysplasia is a neurodevelopmental disorder characterized by disorganization of the cortical layers in the cerebellum. Much of the early research focused on the neuronal component in cortical dysplasia; however, it is obvious to expect the involvement of BG and astrocytes in its pathology. Rodríguez-Arzate et al. reported several functional and morphological changes in BG and astrocytes in the animal model of cerebellar cortical dysplasia [4]. The authors found dysfunctional maturation and development of BG and astrocytes leading to less cellular complexity and altered intracellular calcium oscillations. Using many advanced techniques, the authors provided evidence that glial cells are a possible source of disrupted synaptic transmission, which can be further investigated to better understand the pathological basis of cerebellar cortical dysplasia.

Retinal Müller glial (RMG) cells are the principal glial cells of the retina and actively participate in maintaining water and ion homeostasis in the retina. Additionally, RMG cells are very responsive to pathogenic stimuli and undergo extensive morphological and biochemical changes. These reactive RMG cells have been identified in the neuroinflammatory environment of different retinal diseases. The study by Lorenz et al. showed the potential immune-related responses of RMG cells after lipopolysaccharide (LPS) stimulation [5]. Mass spectrometric analysis of the RMG surfaceome (surface proteome) after LPS stimulation showed the enrichment of many immune-related pathways. A notably higher abundance of proteins related to immune signal detection, antigen presentation, and cell adhesion was found in LPS-stimulated RMG, pointing toward the potential neuroinflammatory role of RMG in ocular diseases. The authors also found novel proteins associated with immune cell migration and infiltration. These findings aid our understanding of RMG biology, leading to the identification of the stimuli-dependent neuroimmune capacity of RMG and their contribution to neuroinflammatory signaling in the retina.

Neuroinflammation is a shared feature of different CNS pathologies, which is regulated mainly by specialized brain immune and inflammatory cells, including astrocytes and microglia. The molecular mechanisms and signaling pathways involved in inflammatory activation and diverse neuroimmune responses of microglia and astrocytes are thought to be context-dependent and are yet to be fully disentangled. Using genetic interaction screening in a yeast model expressing a mutant form of human transactive response DNA-binding protein 43 (TDP-43), Kim et al. identified crucial interacting partners of TDP-43 that play an essential role in the inflammatory activation of astrocytes [6]. Intranuclear and cytoplasmic TDP-43 inclusions in motor neurons have been identified as a common pathological characteristic of amyotrophic lateral sclerosis (ALS) and frontotemporal dementia (FTD). More recently, glial TDP-43 inclusions have received attention, as glial activation precedes the onset of neurodegeneration in ALS and FTD pathology. The study by Kim et al. highlights the glial aspect in ALS and FTD, identifying 13 genetic interactors of TDP-43 in astrocytes, including succinate dehydrogenase flavoprotein subunit A (SDHA) and voltage-dependent anion-selective channel 3 (VDAC3), implicated in astrocytic inflammatory activation. The pharmacological SDHA and VDAC3 inhibition in astrocytes bearing mutant forms of TDP-43 decreased inflammatory activation, highlighting diverse mechanisms underlying the inflammatory activation of astrocytes regulated by TDP-43. The newly identified genetic interactions can be exploited as therapeutic targets.

Therapeutic modulation of glial inflammatory activation has exhibited promising effects in numerous neuroinflammatory conditions. In their study, Yamashita et al. exploited the same concept—‘inhibition of inflammatory activation of microglia’—in the neuropathic pain model [7]. The authors screened a chemical library of clinically approved drugs for their inhibitory effects on microglial ATP-gated P2X7 receptors (P2X7R) and identified the calcium channel blocker cilnidipine as a potent inhibitor of P2X7R, which can be exploited as a possible analgesic agent in conditions presenting with neuropathic pain as one of the major clinical symptoms.

This SI also includes a series of review articles highlighting the roles of various types of glia in regulating CNS homeostasis and their potential contribution to disease conditions. The existence of structural and functional diversity within the glial population explains the diverse roles played by these cells in a region-specific manner. In their review, Buzoianu-Anguiano et al. shed light on molecular and functional aspects of a group of glial cells, collectively named aldynoglia [8]. They further extended their discussion to the regenerative potential of aldynoglia in spinal cord injury and suggested many mechanisms by which these cells can be used as therapeutics in spinal cord injury.

The review by Ghasemi et al. covers the chromosome 9 open reading frame 72 (*C9orf72*) mutation-induced pathological mechanisms in ALS and FTD [9]. The authors elegantly summarized the literature advocating for *C9orf72* mutation-induced glial neurotoxicity in ALS and FTD pathology. Glial cell activation is positively correlated with neurodegeneration in *C9orf72*-related ALS and FTD and thus appears to be a fascinating avenue for further investigation for clinical translation. The last review of this Special Issue by Hwang et al. comprehensively discussed astrocytes’ roles in regulating diverse behaviors through neuronal modulation [10]. The region-specific regulation of neural circuits by astrocytes and the resultant behavior were discussed.

The articles included in the current SI add much to our comprehension of the involvement of glia in the pathophysiological mechanisms of different CNS diseases. The research articles in this SI have employed a range of in vitro- and in vivo-based experiments that hold the potential for clinical implications. The breadth of the work presented here will enhance our understanding of the neuroimmunological roles of glia in diverse neuropathology, with the hope that these findings may pave the way for the discovery and development of therapeutic strategies for treating these devastating disorders.

## Data Availability

Not applicable.
